# Knowledge of pediatricians regarding physical activity in childhood and adolescence

**DOI:** 10.1016/j.rpped.2015.02.001

**Published:** 2015

**Authors:** Alex Pinheiro Gordia, Teresa Maria Bianchini de Quadros, Luciana Rodrigues Silva, Gilton Marques dos Santos

**Affiliations:** a Universidade Federal do Recôncavo da Bahia (UFRB), Amargosa, BA, Brazil; b Universidade Federal da Bahia (UFBA), Salvador, BA, Brazil

**Keywords:** Motor activity, Education medical, Pediatric obesity, Health promotion

## Abstract

**Objective:**

To investigate the knowledge and guidance given by pediatricians regarding physical activity in childhood and adolescence.

**Methods:**

A cross-sectional study involving a convenience sample of pediatricians (*n*=210) who participated in a national pediatrics congress in 2013. Sociodemographic and professional data and data regarding habitual physical activity and pediatricians’ knowledge and instructions for young people regarding physical activity were collected using a questionnaire. Absolute and relative frequencies and means and standard deviations were calculated.

**Results:**

Most pediatricians were females, had graduated from medical school more than 15 years ago, and had residency in pediatrics. More than 70% of the participants reported to include physical activity guidance in their prescriptions. On the other hand, approximately two-thirds of the pediatricians incorrectly reported that children should not work out and less than 15% answered the question about physical activity barriers correctly. With respect to the two questions about physical activity to tackle obesity, incorrect answers were marked by more than 50% of the pediatricians. Most participants incorrectly reported that 30 min should be the minimum daily time of physical activity in young people. Less than 40% of the pediatricians correctly indicated the maximum time young people should spend in front of a screen.

**Conclusions:**

In general, the pediatricians reported that they recommend physical activity to their young patients, but specific knowledge of this topic was limited. Programs providing adequate information are needed.

## Introduction

Obesity in childhood and adolescence has been considered a pandemic, with high costs for health care systems worldwide.^1^ Young obese individuals are more likely to develop cardiometabolic risk factors, diabetes, hypertension, liver disease, joint disease, asthma, oral health problems, anxiety, depression, attention deficit disorders with hyperactivity, sleep problems and negative perception of quality of life.^2^Obesity during childhood and adolescence has adverse effects on early mortality and physical morbidity in adulthood in the short and long terms.^3^

Evidence indicates that physical activity during childhood and adolescence may contribute to fight obesity in at least three ways: (1) the practice of physical activity in childhood and adolescence helps maintaining the energy balance and, consequently, aids in the prevention and treatment of obesity and obesity-related diseases in this stage of life; (2) active young individuals tend to become active adults, increasing energy expenditure throughout the life cycle, and (3) active young individuals are less likely to develop obesity and obesity-related diseases in adulthood.^4^^,^ 5 However, while physical activity represents an important component of health promotion and disease prevention in children,^5^^,^ 6 there is a high prevalence of sedentary behavior and insufficient physical activity in this population.^7^^,^ 8

Pediatricians are the health professionals more often present in young individuals’ lives, accompanying them from birth to late adolescence. In this sense, pediatricians are very important to fight the obesity pandemic in children with a focus on health promotion, protection, prevention and health education. Parents believe that pediatricians have a central role in controlling the body weight of their children, including the suggestion of diet plans and physical activity.^9^ As the family is the primary source of information and support to the pediatric patient, providing patient and family-centered care is essential for the success of pediatric practice.^10^ There is evidence that young individuals who have parental support for physical activity are more active, as well as that more active parents have more active children.^11^Thus, sporadic information about physical activity given by the pediatrician, both to the patient and to their parents, may be a promising strategy for adherence to this practice at an early age.

However, there is still little information about the training and the role of the pediatrician as a physical activity promoter. Therefore, the aim of this study was to investigate the knowledge and information provided by pediatricians on physical activity during childhood and adolescence.

## Method

Cross-sectional, descriptive study, carried out with a convenience sample of physicians who participated in the VII *Congresso Baiano de Pediatria* (Pediatrics Conference) in Salvador, Bahia, or in the VI *Congresso Gaúcho de Atualização em Pediatria* (Conference of Pediatrics Update), held in Porto Alegre, Rio Grande do Sul, both in 2013. There were 256 registered physicians in the VII *Congresso Baiano de Pediatria* and 310 in the VI *Congresso Gaúcho de Atualização em Pediatria*. All physicians registered in the conferences received a questionnaire in their folders. On two occasions during the conferences, participants received information about the study and were asked to complete the questionnaire. At the end of each activity carried out at the events, there was a researcher responsible for collecting the filled out questionnaires. Although not all investigated physicians had done residency in Pediatrics, in this study we chose to call all of them "pediatricians" because of their practical work in this area.

As an evaluation tool, a questionnaire that was formulated by two teachers of physical education and a pediatrician was used. The tool consisted of 20 questions, divided into two parts. The first part, consisting of seven questions, investigated the information about gender, time since graduation from the medical school, residency in Pediatrics, time working in Pediatrics, workplace (outpatient clinic/private clinic, hospital ward, emergency room and more than one location) and employment sector (only public, only private or both sectors). Still in the first part of the questionnaire, the participants were asked about their usual physical activity practice with the following question: "On average, how much time in a typical week do you dedicate to physical activity?" The second part was structured in two other parts: (1) knowledge about physical activity in childhood and adolescence (ten questions), and (2) guidelines/recommendations for children, adolescents and parents about physical activity (three questions). The tool was developed based on the American College of Sports Medicine guidelines^12^ and other classical references of the area.^13^^-^ 15

Data were analyzed using SPSS statistical software, version 20.0. Absolute and relative frequencies, mean and standard deviation (SD) were calculated. Prior to participation in the study, pediatricians were informed about the research objectives and voluntarily agreed to participate. The present study was approved by the Institutional Review Board of Faculdade Maria Milza (process #126/2011).

## Results

The study included 210 pediatricians (145 in the event in Salvador, BA, and 65 in Porto Alegre, RS). There was a sample loss of 356 pediatricians, with 111 in the event held in Salvador, BA and 245 in the event held in Porto Alegre, RS. Pediatricians reported practicing 105.5 (SD=60.8) minutes of physical activity on a typical week. The demographic and professional data of the assessed pediatricians are shown in Table 1. Most of them were females, had graduated from medical school more than 15 years before, had done residency in Pediatrics and worked with Pediatrics for more than 15 years. The majority of pediatricians worked in an outpatient clinic/private clinic or at more than one location, and more than half worked in both the public and private sectors.

**Table 1:** Demographic and professional characteristics of the interviewed pediatricians.

Characteristic	*n* (Total)	%
*Gender*		
Male	62 (206)	20.4
Female	164 (206)	79.6

*Time since Medical School graduation (years)*		
≤5	35 (205)	17.1
6–15	36 (205)	17.6
16–25	48 (205)	23.3
≥26	86 (205)	42.0

*Medical residency in Pediatrics*		
Yes	168 (203)	82.8
No	35 (203)	17.2

*Time working in Pediatrics (years)*		
≤5	43 (194)	22.2
6–15	32 (194)	16.5
16–15	52 (194)	26.8
≥26	67 (194)	34.5

*Place of employment*		
Outpatient Clinic/private practice	84 (198)	42.5
Nursing ward	3 (198)	1.5
Emergency Room	22 (198)	11.1
More than one place of employment	89 (198)	44.9

*Work Sector*		
Only public sector	44 (194)	22.7
Only private sector	42 (194)	21.6
Both sectors	108 (194)	55.7

More than 90% of respondents gave a correct answer in that physical activity recommendations for children should not be similar to those for adults, and that recreational activities are more suitable than competitive activities for children. On the other hand, more than two thirds incorrectly reported that children cannot perform strength training, and less than 15% correctly answered the question about impediments to physical activity. Many of the assessed pediatricians reported that they did not know the American College of Sports Medicine's recommendations for physical activity in the pediatric population (Table 2).

**Table 2:** Frequency of answers on the general aspects of physical activity in childhood and adolescence.

Questions/answers	*n* (Total)	%
*Do you know the American College of Sports Medicine's recommendations for the practice of physical activities of children and adolescents?*		
Yes	32 (197)	16.2
No	165 (197)	83.8

*Considering physiological aspects such as thermoregulation, aerobic and anaerobic metabolism, should the recommendations for practice of physical activities by children be similar to the recommendations for adults?*		
Yes	5 (191)	2.6
No^a^	186 (191)	97.4

*What activities are more adequate for children?*		
Competitive	11 (187)	5.9
Recreational^a^	176 (187)	94.1
*Can children and adolescents do strength training?*		
Yes^a^	52 (188)	27.7
No	136 (188)	72.3

*Which of the following options is not a significant impediment to physical activity in children and adolescents? Check only one.*		
Lack of time	61 (184)	33.2
Lack of family support	17 (184)	9.3
Fear of hurting oneself^a^	26 (184)	14.1
Lack of opportunity	21 (184)	11.4
Laziness	47 (184)	25.5
Lack of safety	12 (184)	6.5

a Correct answer.

In both questions on physical activity to fight obesity in childhood and adolescence, at least half of pediatricians indicated incorrect alternatives. Many respondents would recommend the practice of isolated aerobic activities and walking as the most appropriate physical activity for obese young individuals (Table 3).

**Table 3:** Frequency of answers on practice of physical activities to fight obesity in childhood and adolescence.

Questions/answers	*n* (Total)	%
*For obese children and adolescents, what physical activity would you recommend to help weight loss?*		
Aerobic activities	102 (191)	53.4
Anaerobic activities	11 (191)	5.8
Both^a^	78 (191)	40.8

*For obese children and adolescents, considering the most appropriate type of physical activity, which option would you recommend the most? Check only one.*		
Walking	81 (186)	43.5
Running	8 (186)	4.3
Swimming^a^	93 (186)	50.0
Volleyball	0 (186)	0
Soccer	4 (186)	2.2

a Correct answer.

Most of the assessed pediatricians incorrectly reported that 30 min is the minimum daily time for physical activity for children and adolescents. In addition, less than 40% were able to correctly answer what is the maximum time that young individuals can spend in front of screens, and almost half did not identify active video games as helpers in increasing the level of physical activity in the pediatric population (Table 4).

**Table 4:** Frequency of answers on active and sedentary behavior in childhood and adolescence.

Questions/answers	*n* (Total)	%
*What should be the minimum daily duration of time spent on physical activity for children and adolescents according to the American College of Sports Medicine's recommendations?*		
30 min	109 (177)	61.6
45 min	25 (177)	14.1
60 min^a^	38 (177)	21.5
75 min	0 (177)	0
90 min	5 (177)	2.8

*What should be the maximum daily duration of time spent by children and adolescents in front of screens (TV, video game and computer) according to the current recommendations?*		
15 min	2 (183)	1.1
30 min	28 (183)	15.3
60 min	73 (183)	39.9
90 min	9 (183)	4.9
120 min^a^	71 (183)	38.8

*Can active video games (such as Nintendo Wii) help to increase the physical activity of children and adolescents?*		
Yes^a^	101 (185)	54.6
No	84 (185)	45.4

a Correct answer.

Most of the assessed pediatricians reported incorporating information on physical activity in their prescriptions and offering guidance to parents of obese young individuals about the importance of a lifestyle change in the whole family. In addition, over 60% reported recommending that their young patients seek a physical education professional for the practice of specific physical activities (Table 5).

**Table 5:** Frequency of answers on information and recommendations by pediatricians for physical activity in childhood and adolescence.

Questions/answers	*n* (Total)	%
*Do you incorporate the recommendations of physical activity in your systematic prescriptions?*		
Yes	137 (187)	73.3
No	50 (187)	26.7
Only for obese children	0 (187)	0

*In your practice, do you have the habit of making recommendations to the parents of young obese patients about the importance of lifestyle changes in the whole family?*		
Yes	185 (190)	97.4
No	5 (190)	2.6

*In your practice, do you recommend that young patients seek a physical education professional for the practice of specific physical activities?*		
Yes	121 (190)	63.7
No	69 (190)	36.3

## Discussion

The increase in the regular practice of physical activity in childhood and adolescence has been reported as an effective and promising strategy to fight obesity and, consequently, to decrease chronic, noncommunicable diseases in adults.^4^^-^ 6 Together with other health professionals, pediatricians are considered to be facilitators of this process, as they are the first professional to be sought by caregivers, as they accompany the child throughout the growth process and because of the confidence that parents have in their work. In this study, we observed that most of the investigated pediatricians recommended and provided information on physical activity during their clinical practice. However, the assed pediatricians usually had limited knowledge on the subject. Most of them mistook recommendations for the adult population as adequate for children, reported that children cannot do strength training, were unaware of the current recommendations on the maximum daily time allowed for children in front of screens and erroneously indicated the most appropriate activities for obese children and adolescents.

Recommendations and guidelines for the practice of physical activity in children and adolescents should take into account specific physiological responses that occur during exercise, especially in the thermoregulation and energy production systems.^16^^-^ 18 Children have less sweat output by gland, and thus absorb more heat during exercise under heat stress, a characteristic that can lead them to have symptoms of hyperthermia.^16^ Additionally, children have a higher body surface per unit of body mass than adults, which increases the speed of heat exchange with the environment, accelerating both the heat gain in hot climates (greater risk for hyperthermia) and heat loss in cold climates (higher risk for hypothermia).^16^^,^ 18Therefore, it is important that young individuals exercise in thermoneutral environments and are properly hydrated before, during and after practice.^12^ Regarding energy production, when compared to adults, children have lower levels of lactate dehydrogenase and phosphofructokinase enzymes, which limit the performance of anaerobic activities, and have lower systolic volume and cardiac output, which reduce the capacity to perform aerobic activities.^16^^,^ 17 This immaturity of thermoregulation and energy production systems is evident in prepubertal individuals and tends to decrease during puberty. Thus, from a physiological point of view, at the end of adolescence the recommendations and guidelines for the practice of physical activity may be similar to those of adults.

According to the current literature,^12^^,^ 19 ^,^ 20 children and adolescents should be urged to practice at least 60 min of moderate to vigorous physical activity a day, whereas for adults, it is recommended a minimum of 30 min a day.^12^ The adult recommendation was established nearly 20 years ago^21^ and, since then, it has been widely disseminated by the mass media. In this study, over 60% of the assessed pediatricians indicated the suggested 30 min a day for adults as the minimum amount of physical activity to be recommended for children and adolescents. This finding is of concern, because young individuals can be encouraged to perform only half of the recommended daily time of physical activity, with a significant reduction in health benefits. Public policies and civil society campaigns aimed at increasing the dissemination of recommendations for physical activity in childhood and adolescence, both for health professionals and for the general population, may be useful to promote this behavior in children, emphasizing that the ideal duration of physical activity is at least 60 min a day, every day of the week.

Physical activities for children and adolescents should be nice and appropriate for growth/development and may include walking, active play/games, dance, sports and activities to strengthen muscles and bones.^12^Another important aspect about the prescription and physical activity information for young individuals are the factors that hinder or create difficulties for the practice. Evidence indicates that "lack of time", "lack of family support," "lack of company of friends", "lack of opportunity", "prefer to do other things," "laziness", "do not have anyone to take me", "lack of space" and "lack of safety" have been considered impediments to physical activity during childhood and adolescence.^15^^,^ 22 The knowledge of these impediments by pediatricians and the attempt to propose strategies for young individuals to try overcoming them may be relevant to increasing physical activity.

Although there is a consensus in the literature that activities aiming at muscle strength/endurance gaining, such as resistance training, are beneficial to children and adolescents,^23^ this practice is still seen with a lot of prejudice or synonymous of an exclusively adult activity. This bias emerged in the 1970s and 1980s, when a survey in the US on the number of lesions in young practitioners of strength/resistance activities was performed. However, the lesions were related to problems in the equipment ergonomics, lack of supervision and planning by a trained professional and excess workload.^23^ Current studies on strength training/muscular endurance indicate that, when an appropriate plan is followed, this practice can be safe, effective, enjoyable and with low risk of injury.^12^^,^ 19 ^,^ 20 ^,^ 23 According to the American College of Sports Medicine^12^ and other guidelines,^19^^,^ 20 children and adolescents should perform strength/resistance activities for the major muscle groups 2-3 times a week, with 2-4 series per session and 8-15 repetitions per series, with moderate workload and focus on improvement of the motor gesture. Muscular strength/endurance activities, if properly prescribed and supervised, besides offering less risk of osteoarticular lesions in children than contact sports,^18^ provide significant gain in bone density and mineral content.^19^^,^ 23 Additionally, muscular strength/muscular resistance activities can help in the improvement of cardiorespiratory fitness and body composition and, therefore, reduce cardiovascular risk factors.^23^

Physical activity plays an important role in obesity prevention and treatment in the pediatric population.^4^^,^ 5 A systematic review of meta-analyzes demonstrated that regular physical activity was effective in reducing the percentage of body fat in obese young individuals.^5^Both aerobic (e.g. cycling, walking and swimming) and anaerobic (e.g. strength training) activities are important for the obesity treatment. Aerobic activities are recommended for weight loss in obese individuals due to the high energy expenditure and fat metabolism stimulation, whereas anaerobic activities are suggested to assist in maintaining the basal metabolic rate by preserving lean mass during body weight loss.^24^^,^ 25 However, it is important to bear in mind that obese young individuals feel more pain in the knees, have greater impairment of mobility and higher prevalence of fractures and joint and musculoskeletal discomfort, compared to individuals with normal weight.^26^ In this sense, physical activity programs for this part of the population should include exercises that require little or no joint impact. The American College of Sports Medicine^12^ suggests that training for obese individuals emphasize activities without support of body weight, such as resistance training, riding a stationary bike, swimming and exercises in the water. Among the aerobic activities, swimming and exercises in the water deserve special attention, as they are dynamic activities that allow high energy expenditure because they are performed in a nice/attractive environment (water), by showing relevant adherence by obese young individuals, because of the low risk of lesions and because they allow the activation of muscle groups in the upper and lower limbs and trunk.^27^^,^ 28

Sedentary behavior may be a risk factor for adverse effects on health even in young individuals who meet the recommendations for physical activity.^29^ It is suggested that children and adolescents do not exceed 2 h a day in sedentary activities, in order to prevent obesity and cardiovascular risk factors.^13^ The access of the pediatric population to television, cell phones, video games and computers has increased in recent years and, with that, many young individuals spend a long time in front of screens.^8^ On the other hand, technological advances can also help to increase the physical activity practice of young individuals through active video games. A systematic review has shown that these games can increase levels of physical activity, energy expenditure, maximum oxygen consumption and heart rate, and decrease waist circumference and sedentary time in front of screens.^30^ Considering that electronic media tend to be increasingly present in the daily lives of young individuals, it is suggested that the pediatrician's information to the patient aim to limit at 2-h a day the time spent by young individuals in sedentary activities in front of screens, and strongly encourage the use of active video games as a strategy to increase physical activity.

The present study has limitations, including the convenient sample of physicians who participated in the two pediatric update events, as well as the sampling loss of 62.9% of the study population. Therefore, the study's external validity and extrapolation of findings become limited. Regarding the internal validity of the study, the absence of sample calculation and sampling strategy can be a limitation, considering the high sample loss. However, the questionnaire used as a tool for data collection was not validated and might not determine the true significance of what we proposed to measure. The evaluation of the knowledge and information provided by the pediatricians participating in the conferences exclusively through a questionnaire makes it impossible to determine if what was reported is what actually happens in the daily clinical practice of those professionals in outpatient clinics and offices, during short-time consultations. Despite these issues, this study demonstrates that the knowledge and information provided by pediatricians still need improvement regarding the appropriate physical activity for children and adolescents.

In general, the assessed pediatricians reported they recommend physical activity for their young patients, but the specific knowledge on the subject was limited. Therefore, the role of the pediatrician as a facilitator for the adherence of young individuals to physical activity is compromised. In addition to supporting physical activity, especially to fight obesity, pediatricians, due to their privileged contact with young individuals and their families, have also been asked to give advice on healthy eating, oral health, mental health, vaccination, among others. Based on this context, three questions seem to arise: (1) Are pediatricians responsible for informing their patients on all aspects related to health promotion of children and adolescents?; (2) Do graduate courses in medicine and Pediatric residency have curricula with theoretical and practical basis to provide support for pediatricians to safely include these guidelines in their clinical practice?, and (3) Is there an effective opportunity for the pediatrician's interaction with other primary health care professionals, such as physical educators, nutritionists, dentists and psychologists, in the comprehensive care given to children and adolescents? The reflection, discussion and development of research based on these questions may have consequences that will help in the development of policies based on new models of training and performance of pediatricians and other professionals who participate in the monitoring process of young individuals’ health, and in providing adequate information on healthy life habits.
