# Supplementary material for: Knowledge of pediatricians regarding physical activity in childhood and adolescence
Source: Rev Paul Pediatr. 2015 Oct-Dec;33(4):400–6. doi: 10.1016/j.rpped.2015.02.001 (PMC4685559; doi:10.1016/j.rpped.2015.02.001)
Supplement: Translation

# Translation: Conhecimento de pediatras sobre a atividade física na infância e adolescência

**Authors:** Alex Pinheiro Gordia, Teresa Maria Bianchini de Quadros, Luciana Rodrigues Silva, Gilton Marques dos Santos

**Affiliations:** a Universidade Federal do Recôncavo da Bahia (UFRB), Amargosa, BA, Brasil; b Universidade Federal da Bahia (UFBA), Salvador, BA, Brasil

**Keywords:** Atividade motora, Educação médica, Obesidade pediátrica, Promoção da saúde

## Abstract

**Objetivo:**

Investigar o conhecimento dos pediatras sobre a atividade física na infância e adolescência e a orientação dada por eles.

**Métodos:**

Estudo transversal feito com uma amostra conveniente de pediatras (n=210) que participaram de congressos nacionais destinados à especialidade em 2013. Usou-se um questionário para coletar dados sociodemográficos, profissionais, da prática habitual de atividade física e sobre o conhecimento e orientação do pediatra em relação à atividade física de jovens. Foram calculadas frequências absolutas e relativas, média e desvio-padrão.

**Resultados:**

A maior proporção dos pediatras era do sexo feminino, estava formado em medicina havia mais de 15 anos e tinha residência em pediatria. Mais de 70% relataram incorporar a orientação de atividade física em suas prescrições. Por outro lado, aproximadamente dois terços relataram incorretamente que crianças não podem fazer musculação e menos de 15% responderam corretamente a questão sobre barreiras para a prática de atividade física. Em ambas as questões sobre a prática de atividade física para enfrentamento da obesidade as opções incorretas foram marcadas por 50% ou mais dos pediatras. A maioria relatou, incorretamente, que 30 minutos é o tempo mínimo diário para a prática de atividade física para jovens. Menos de 40% souberam responder corretamente qual é o tempo máximo que os jovens podem passar em frente às telas.

**Conclusões:**

Em geral, os pediatras relataram recomendar a atividade física para seus pacientes jovens, porém o conhecimento específico sobre o assunto foi limitado. Há necessidade de programas com informações adequadas para os pediatras.

## Introdução

A obesidade na infância e adolescência tem sido considerada uma pandemia, com elevados custos para os sistemas de cuidado à saúde em todo o mundo.^1^ Jovens obesos apresentam maior probabilidade de desenvolver fatores de risco cardiometabólicos, diabetes, hipertensão, hepatopatia, doença articular, asma, problemas de saúde bucal, ansiedade, depressão, transtornos de déficit de atenção com hiperatividade, problemas de sono e percepção negativa de qualidade de vida.^2^ A obesidade durante a infância e adolescência tem consequências adversas sobre a mortalidade precoce e morbidade física na idade adulta em curto e longo prazo.^3^

Evidências indicam que a atividade física durante a infância e a adolescência pode contribuir para o enfrentamento da obesidade ao menos por três caminhos: 1) a prática de atividade física na infância e adolescência auxilia no equilíbrio do balanço energético e, consequentemente, na prevenção e no tratamento da obesidade e de doenças relacionadas à obesidade nessa fase da vida; 2) jovens ativos tendem a se tornar adultos ativos, a aumentar o gasto energético durante todo o ciclo de vida; e 3) jovens ativos têm menor probabilidade de desenvolver obesidade e doenças relacionadas à obesidade na fase adulta.^4^^,^ 5 Contudo, ainda que a atividade física represente um importante componente da promoção da saúde e prevenção de doenças na população pediátrica,^5^^,^ 6 observa-se elevada prevalência de comportamentos sedentários e prática insuficiente de atividade física nesse segmento populacional.^7^^,^ 8

O pediatra é o profissional de saúde mais presente na vida dos jovens, acompanha-os desde o nascimento até o fim da adolescência. Nesse sentido, o pediatra pode ser muito importante para o enfrentamento da pandemia da obesidade na população infantil com foco na promoção, proteção, prevenção e educação para a saúde. Os pais acreditam que os pediatras têm papel central no controle de peso corporal de seus filhos, incluindo a sugestão de planos de dieta e de prática de atividades físicas.^9^ Como a família é a fonte primária de informação e apoio para o paciente pediátrico, fornecer cuidados centrados no paciente e na família é essencial para o sucesso da prática pediátrica.^10^ Há evidências de que jovens com apoio dos pais para prática de atividades físicas são mais ativos, assim como pais mais ativos têm filhos mais ativos.^11^ Assim, a orientação periódica do pediatra sobre a atividade física, tanto ao paciente quantos aos seus pais, pode ser uma estratégia promissora para a adesão a essa prática em idades precoces.

No entanto, ainda são escassas as informações sobre a capacitação e a atuação do pediatra como promotor da atividade física. Dessa forma, o objetivo do presente estudo foi investigar o conhecimento dos pediatras sobre a atividade física durante a infância e a adolescência e a orientação dada por eles.

## Método

Estudo transversal, descritivo, feito com uma amostra de conveniência de médicos que participaram do VII Congresso Baiano de Pediatria em Salvador, BA, ou do VI Congresso Gaúcho de Atualização em Pediatria em Porto Alegre, RS, ambos em 2013. Havia 256 médicos inscritos no VII Congresso Baiano de Pediatria e 310 no VI Congresso Gaúcho de Atualização em Pediatria. Todos receberam um questionário. Em duas oportunidades, durante os congressos, os participantes receberam informações sobre o estudo e foram convidados a responder o questionário. No fim de cada atividade nos eventos, havia um pesquisador responsável pela coleta dos questionários respondidos. Ainda que nem todos os médicos investigados tivessem residência em pediatria, no presente estudo optou-se por denominar todos como "pediatras" em virtude da atuação prática nessa área.

Como instrumento de avaliação foi usado um questionário formulado conjuntamente por dois professores de educação física e uma pediatra. O instrumento foi composto por 20 questões, divididas em duas partes. A parte inicial, composta por sete questões, investigou informações sobre o sexo, tempo de formado, residência médica em pediatria, tempo de trabalho em pediatria, local de trabalho (ambulatório/consultório, enfermaria, emergência e mais de um local) e setor de trabalho (somente público, apenas privado e ambos os setores). Ainda na parte inicial os participantes foram perguntados sobre sua prática habitual de atividades físicas: "Em média, quanto tempo em uma semana típica você dedica para a prática de atividades físicas?" A segunda parte foi estruturada em dois blocos: 1) conhecimento sobre a atividade física na infância e adolescência (com dez questões) e; 2) orientações/recomendações para crianças, adolescentes e pais sobre a prática de atividade física (com três questões). O instrumento foi desenvolvido com base nas diretrizes do Colégio Americano de Medicina Esportiva^12^ e em outras referências clássicas da área.^13^^-^ 15

Os dados foram analisados no programa estatístico SPSS, versão 20.0. Foram calculadas frequências absolutas e relativas, média e desvio-padrão (dp). Previamente à participação no estudo, os pediatras foram informados sobre os objetivos da investigação e aceitaram participar voluntariamente. O protocolo do presente estudo foi aprovado pelo Comitê de Ética em Pesquisa da Faculdade Maria Milza (processo n°126/2011).

## Resultados

Participaram do estudo 210 pediatras (145 do evento em Salvador, BA, e 65 em Porto Alegre, RS). Houve perda amostral de 356, 111 do evento em Salvador, BA, e 245 do evento em Porto Alegre, RS. Os pediatras relataram praticar 105,5 (dp=60,8) minutos de atividades físicas em uma semana típica. As informações demográficas e profissionais dos pediatras investigados podem ser observadas na Tabela 1. A maior parte era do sexo feminino, estava formada em medicina havia mais de 15 anos, tinha residência em pediatria e trabalhava com pediatria havia mais de 15 anos. Grande proporção dos pediatras trabalhava em ambulatório/consultório ou em mais de um local e mais da metade trabalhava tanto no setor público quanto no privado.

**Tabela 1:** Características demográficas e profissionais dos pediatras entrevistados

Característica	n (Total)	%
*Sexo*		
asculino	62 (206)	20,4
Feminino	164 (206)	79,6

*Tempo de formado em medicina (anos)*		
≤5	35 (205)	17,1
6‐15	36 (205)	17,6
16‐25	48 (205)	23,3
≥26	86 (205)	42,0

*Residência médica em pediatria*		
Sim	168 (203)	82,8
Não	35 (203)	17,2

*Tempo de trabalho em pediatria (anos)*		
≤5	43 (194)	22,2
6‐15	32 (194)	16,5
16‐15	52 (194)	26,8
≥26	67 (194)	34,5

*Local de trabalho*		
Ambulatório/consultório	84 (198)	42,5
Enfermaria	3 (198)	1,5
Emergência	22 (198)	11,1
Mais de um local de trabalho	89 (198)	44,9

*Setor de trabalho*		
Apenas no setor público	44 (194)	22,7
Apenas no setor privado	42 (194)	21,6
Em ambos os setores	108 (194)	55,7

Mais de 90% dos entrevistados responderam corretamente que as recomendações para a prática de atividades físicas de crianças não devem ser semelhantes às de adultos e que atividades recreativas são mais adequadas do que atividades competitivas para crianças. Por outro lado, mais de dois terços relataram incorretamente que crianças não podem fazer musculação e menos de 15% responderam corretamente a questão sobre barreiras para prática de atividades físicas. Grande parte dos investigados relatou não conhecer as recomendações do Colégio Americano de Medicina Esportiva para a prática de atividades físicas da população pediátrica (Tabela 2).

**Tabela 2:** Frequência de respostas sobre aspectos gerais da atividade física na infância e adolescência

Questões/respostas	n (Total)	%
*Você conhece as recomendações do Colégio Americano de Medicina Esportiva para a prática de atividades físicas de crianças e adolescentes?*		
Sim	32 (197)	16,2
Não	165 (197)	83,8

*Considerando aspectos fisiológicos como a termorregulação, o metabolismo aeróbio e o anaeróbio, as recomendações para a prática de atividades físicas de crianças devem ser semelhantes às recomendações para adultos?*		
Sim	5 (191)	2,6
Não^a^	186 (191)	97,4

*Que atividades são mais adequadas para crianças?*		
Competitivas	11 (187)	5,9
Recreativas^a^	176 (187)	94,1

*Crianças e adolescentes podem fazer musculação?*		
Sim^a^	52 (188)	27,7
Não	136 (188)	72,3

*Qual das opções não é uma barreira relevante para prática de atividade física de crianças e adolescentes? Marque apenas uma.*		
Falta de tempo	61 (184)	33,2
Falta de apoio familiar	17 (184)	9,3
Medo de se machucar^a^	26 (184)	14,1
Falta de oportunidade	21 (184)	11,4
Preguiça	47 (184)	25,5
Falta de segurança	12 (184)	6,5

a Resposta correta.

Em ambas as questões sobre a prática de atividades físicas para enfrentamento da obesidade na infância e adolescência, ao menos metade dos pediatras assinalou opções incorretas. Grande parte dos respondentes recomendaria a prática de atividades aeróbias isoladas e a caminhada como atividade física mais adequada para jovens obesos (Tabela 3).

**Tabela 3:** Frequência de respostas sobre a prática de atividades físicas para enfrentamento da obesidade na infância e adolescência

Questões/respostas	n (Total)	%
*Para crianças e adolescentes obesos, qual atividade física você recomendaria para auxiliar na perda de peso?*		
Atividades aeróbias	102 (191)	53,4
Atividades anaeróbias	11 (191)	5,8
Ambas^a^	78 (191)	40,8

*Para crianças e adolescentes obesos, considerando o tipo de atividade física mais apropriada, qual das opções você mais recomendaria? Marque apenas uma.*		
Caminhada	81 (186)	43,5
Corrida	8 (186)	4,3
Natação^a^	93 (186)	50,0
Vôlei	0 (186)	0
Futebol	4 (186)	2,2

a Resposta correta.

A maioria dos investigados relatou, incorretamente, que 30 minutos é o tempo mínimo diário para a prática de atividade física para crianças e adolescentes. Além disso, menos de 40% souberam responder corretamente qual é o tempo máximo que os jovens podem passar em frente às telas e praticamente metade não identifica os jogos ativos de videogame como auxiliadores no aumento da prática de atividades físicas da população pediátrica (Tabela 4).

**Tabela 4:** Frequência de respostas sobre comportamento ativo e sedentário na infância e adolescência

Questões/respostas	n (Total)	%
*Qual deve ser o tempo mínimo diário de prática de atividade física para crianças e adolescentes de acordo com as recomendações do Colégio Americano de Medicina Esportiva?*		
30 min	109 (177)	61,6
45 min	25 (177)	14,1
60 min^a^	38 (177)	21,5
75 min	0 (177)	0
90 min	5 (177)	2,8

*Qual deve ser o tempo máximo diário que crianças e adolescentes deveriam ficar em frente às telas (TV, videogame e computador) segundo recomendações vigentes?*		
15 min	2 (183)	1,1
30 min	28 (183)	15,3
60 min	73 (183)	39,9
90 min	9 (183)	4,9
120 min^a^	71 (183)	38,8

*Jogos ativos de videogame (como do Nintendo Wii) podem auxiliar a aumentar a prática de atividade física de crianças e adolescentes?*		
Sim^a^	101 (185)	54,6
Não	84 (185)	45,4

a Resposta correta.

A maioria dos investigados relatou incorporar a orientação de atividade física nas suas prescrições e orientar os pais de jovens obesos sobre a importância da mudança do estilo de vida de toda a família. Em adição, mais de 60% informaram que recomendam que seus pacientes jovens procurem um profissional de educação física para a prática orientada de atividades físicas (Tabela 5).

**Tabela 5:** Frequência de respostas sobre orientações e recomendações de pediatras para a prática de atividade física na infância e adolescência

Questões/respostas	n (Total)	%
*Você incorpora a orientação de atividade física nas suas prescrições de modo sistemático?*		
Sim	137 (187)	73,3
Não	50 (187)	26,7
Só para obesos	0 (187)	0

*Em sua prática, tem o hábito de orientar os pais de seus pacientes jovens obesos sobre a importância da mudança do estilo de vida de toda a família?*		
Sim	185 (190)	97,4
Não	5 (190)	2,6

*Em sua prática, recomenda que seus pacientes jovens procurem um profissional de educação física para a prática orientada de atividades físicas?*		
Sim	121 (190)	63,7
Não	69 (190)	36,3

## Discussão

O aumento da prática de atividade física habitual na infância e adolescência tem sido relatado como uma estratégia eficaz e promissora no enfrentamento da obesidade e, consequentemente, na diminuição de doenças crônicas não transmissíveis em adultos.^4^^-^ 6 Juntamente com outros profissionais da saúde, o pediatra é considerado facilitador desse processo por ser o primeiro profissional a ser procurado pelos cuidadores, por acompanhar a criança em todo o processo de crescimento e pela confiança que os pais depositam em seu trabalho. Neste estudo foi observado que grande parte dos pediatras investigados recomendava e orientava a atividade física em sua prática clínica. No entanto, os pediatras investigados tinham, em geral, limitado conhecimento sobre o tema. A maioria confundiu recomendações da população pediátrica com as de adultos, relatou que crianças não podem fazer musculação, desconhecia as recomendações vigentes sobre o tempo máximo diário permitido para crianças diante de telas e indicou erroneamente as atividades mais apropriadas para crianças e adolescentes obesos.

As recomendações e orientações para a prática de atividade física de crianças e adolescentes devem levar em consideração respostas fisiológicas específicas que ocorrem durante o exercício, em especial dos sistemas de termorregulação e produção de energia.^16^^-^ 18 Crianças apresentam menor débito de suor por glândula, consequentemente absorvem mais calor durante o exercício sob estresse térmico, característica que pode levá-las a apresentarem sintomas de hipertermia.^16^ Em adição, crianças têm maior superfície corporal por unidade de massa corporal do que adultos, fato que aumenta a velocidade de troca de calor com o meio e acelera tanto o ganho de calor em climas quentes (maior risco de hipertermia) quanto a perda de calor em climas frios (maior risco de hipotermia).^16^^,^ 18 Dessa forma, é importante que jovens exercitem-se em ambientes termoneutros e sejam devidamente hidratados antes, durante e após a prática.^12^ Em relação à produção de energia, quando comparadas com adultos, crianças apresentam menores níveis das enzimas lactatodesidrogenase e fosfofrutoquinase, que limitam atividades anaeróbias, e menor volume sistólico e débito cardíaco, que diminuem a capacidade para atividades aeróbias.^16^^,^ 17 Essa imaturidade dos sistemas de termorregulação e produção de energia são evidentes em pré-púberes e tendem a diminuir durante a puberdade. Logo, do ponto de vista fisiológico, no fim da adolescência as recomendações e orientações para a prática de atividade física podem ser semelhantes àquelas de adultos.

De acordo com a literatura vigente,^12^^,^ 19 ^,^ 20 crianças e adolescentes devem ser instigados a praticar ao menos 60 minutos de atividades físicas moderadas a vigorosas por dia, ao passo que, para adultos, recomenda-se um mínimo de 30 minutos por dia.^12^ A recomendação de adultos foi estabelecida há aproximadamente 20 anos^21^ e, desde então, tem sido amplamente difundida pelos meios de comunicação de massa. No presente estudo, mais de 60% dos pediatras estudados indicou os 30 minutos por dia sugeridos para adultos como a quantidade de atividade física mínima a ser recomendada para crianças e adolescentes. Esse achado é preocupante, pois jovens podem ser encorajados a fazer apenas a metade do tempo diário de atividade física recomendado, com significativa diminuição dos benefícios à saúde. Políticas públicas e campanhas da sociedade que visem a ampliar a divulgação das recomendações para a prática de atividade física na infância e adolescência, tanto para profissionais de saúde quanto para a população em geral, podem ser úteis para promover esse comportamento na população pediátrica e enfatizar que o ideal são no mínimo 60 minutos diários em todos os dias da semana.

As atividades físicas para crianças e adolescentes devem ser agradáveis e apropriadas ao crescimento/desenvolvimento. Podem incluir caminhada, brincadeiras ativas/jogos, dança, esportes e atividades de fortalecimento dos músculos e dos ossos.^12^ Outro aspecto relevante sobre a prescrição e orientação de atividade física para jovens são os fatores que dificultam ou impedem a prática. Evidências indicam que "falta de tempo", "falta de apoio familiar", "falta de companhia dos amigos", "falta de oportunidade", "preferir fazer outras coisas", "preguiça", "não ter alguém para levar", "falta de espaços" e "falta de segurança" têm sido considerados barreiras para a atividade física durante a infância e adolescência.^15^^,^ 22 O conhecimento dessas barreiras por pediatras e a tentativa de propor estratégias para que os jovens tentem superá-las podem ser relevantes para o aumento da prática de atividade física.

Embora haja consenso na literatura de que atividades que visam ao ganho de força/resistência muscular, como a musculação, são benéficas para crianças e adolescentes,^23^ essa prática ainda é vista com muito preconceito ou sinônimo de atividade exclusiva de adultos. Esse preconceito surgiu nas décadas de 1970 e 1980, quando foi feito um levantamento nos EUA sobre o número de lesões em jovens praticantes de atividades de força/resistência muscular. Contudo, as lesões estavam relacionadas a problemas na ergonomia dos equipamentos, falta de orientação e planejamento por um profissional capacitado e carga de trabalho excessiva.^23^ Estudos atuais sobre o treinamento de força/resistência muscular indicam que, quando seguido um guia apropriado, essa prática pode ser segura, eficaz, agradável e com baixo risco de lesões.^12^^,^ 19 ^,^ 20 ^,^ 23 De acordo com o Colégio Americano de Medicina Esportiva^12^ e outros guias,^19^^,^ 20 crianças e adolescentes devem fazer atividades de força/resistência para os principais grupos musculares de duas a três vezes por semana, com duas a quatro séries por sessão e oito a 15 repetições por série, com carga moderada e foco no aperfeiçoamento do gesto motor. As atividades de força/resistência muscular, desde que devidamente prescritas e supervisionadas, além de oferecer menor risco de lesões osteoarticulares em crianças do que os esportes de contato,^18^ proporcionam ganho significativo de densidade e conteúdo mineral ósseo.^19^^,^ 23 Em adição, as atividades de força/resistência muscular podem auxiliar na melhoria da aptidão cardiorrespiratória e composição corporal e, portanto, diminuem os fatores de risco cardiovascular.^23^

A atividade física tem um papel importante para a prevenção e o tratamento da obesidade da população pediátrica.^4^^,^ 5 Revisão sistemática de metanálises demonstrou que a prática regular de atividade física foi eficaz para reduzir o percentual de gordura corporal em jovens obesos.^5^ Tanto as atividades aeróbias (ex.: ciclismo, caminhada e natação) quanto as anaeróbias (ex.: musculação) são importantes para o tratamento da obesidade. Atividades aeróbias são recomendadas para perda de peso corporal de obesos devido ao elevado gasto energético e estímulo ao metabolismo de gorduras, ao passo que atividades anaeróbias são sugeridas para auxiliar na manutenção da taxa metabólica basal por meio da preservação da massa magra durante a perda de peso corporal.^24^^,^ 25 No entanto, é importante ter em mente que jovens obesos sentem mais dor nos joelhos, apresentam maior comprometimento da mobilidade e maior prevalência de fraturas e desconfortos articulares e musculoesqueléticos, em comparação com os eutróficos.^26^ Nesse sentido, programas de atividades físicas para essa parcela da população devem incluir exercícios que exijam pouco ou nenhum impacto articular. O Colégio Americano de Medicina Esportiva^12^ sugere que o treinamento para obesos enfatize atividades sem sustentação do peso corporal, como fazer musculação, andar de bicicleta ergométrica, nadar e fazer exercícios na água. Dentre as atividades aeróbias, a natação e os exercícios na água merecem atenção especial por ser atividades dinâmicas que possibilitam elevado gasto energético, por ser feitas em meio agradável/atrativo (água), por apresentar relevante adesão dos jovens obesos, pelo baixo risco de lesões e por possibilitar a ativação de grupamentos musculares de membros superiores, inferiores e tronco.^27^^,^ 28

O comportamento sedentário pode ser um fator de risco para consequências adversas à saúde mesmo em jovens que atendem às recomendações para a prática de atividades físicas.^29^ Sugere-se que crianças e adolescentes não ultrapassem duas horas por dia em atividades sedentárias para prevenção da obesidade e de fatores de risco cardiovascular.^13^ O acesso da população pediátrica a televisão, celulares, videogames e computadores aumentou nos últimos anos e, com isso, muitos jovens têm permanecido muito tempo em frente a telas.^8^ Por outro lado, o avanço tecnológico também pode contribuir para aumentar a prática de atividade física de jovens por meio de jogos ativos de videogame. Revisão sistemática demonstrou que esses jogos podem aumentar os níveis de atividade física, o gasto de energia, o consumo máximo de oxigênio, a frequência cardíaca, reduzir a circunferência da cintura e o tempo sedentário em frente a telas.^30^ Considerando que as mídias eletrônicas tendem a estar cada vez mais presentes no cotidiano dos jovens, sugere-se que a orientação do pediatra ao paciente objetive limitar em duas horas por dia o tempo destinado pelo jovem a comportamentos sedentários em frente a telas e estimular fortemente o uso de jogos ativos de videogame como estratégia para aumentar a atividade física habitual.

O presente estudo tem limitações, incluindo a amostra conveniente de médicos que participaram de dois eventos de atualização em pediatria, bem como a perda amostral de 62,9% da população de estudo. Dessa forma, a validade externa do estudo e a extrapolação dos achados tornam-se limitadas. No que se refere à validade interna do estudo, a ausência de cálculo e a estratégia amostral podem ser consideradas uma limitação, tendo em vista a elevada perda amostral. Não obstante, o questionário usado como instrumento para coleta de dados não foi validado e pode não determinar o verdadeiro valor do que se propôs a medir. A avaliação do conhecimento e da orientação dos pediatras participantes desses congressos exclusivamente por meio de questionário impossibilita determinar se o que foi relatado é o que efetivamente ocorre na prática clínica diária desses profissionais em ambulatórios e consultórios em tempo exíguo de consultas. Apesar dessas questões, este estudo demonstra que o conhecimento e a orientação dos pediatras ainda necessitam de ampliação nas informações sobre atividade física adequada para crianças e adolescentes.

De forma geral, os pediatras investigados relataram recomendar a atividade física para seus pacientes jovens, porém o conhecimento específico sobre o assunto foi limitado. Nesse sentido, a atuação do pediatra como agente facilitador para a adesão de jovens à prática de atividade física fica comprometida. Além do apoio à prática de atividade física, em especial para o enfrentamento da obesidade, o pediatra, por sua condição privilegiada de contato com os jovens e seus parentes, também tem sido requisitado para dar orientações sobre alimentação saudável, saúde bucal, saúde mental, vacinação, entre outras. Com base nesse contexto, três questões parecem emergir: 1) O pediatra é responsável por orientar seus pacientes sobre todos os aspectos relacionados à promoção da saúde de crianças e adolescentes?; 2) Os cursos de graduação em medicina e residência em pediatria têm currículos com base teórica e prática que garantam apoio para que o pediatra possa incluir, com segurança, essas orientações em sua prática clínica?; e 3) Efetivamente há possibilidade de interação do pediatra com outros profissionais da atenção básica à saúde, como educadores físicos, nutricionistas, dentistas e psicólogos, no cuidado integral à criança e ao adolescente? A reflexão, a discussão e o desenvolvimento de pesquisas sobre essas questões podem ter desdobramentos que auxiliem no direcionamento de políticas baseadas em novos modelos de formação e atuação do pediatra e dos demais profissionais que participam do processo de vigilância à saúde de jovens e a orientação adequada em hábitos de vida saudáveis.
